# Predictors of testing history and new HIV diagnosis among adult outpatients seeking care for symptoms of acute HIV infection in coastal Kenya: a cross-sectional analysis of intervention participants in a stepped-wedge HIV testing trial

**DOI:** 10.1186/s12889-022-12711-1

**Published:** 2022-02-11

**Authors:** Clara A. Agutu, Tony H. Oduor, Amin S. Hassan, Peter M. Mugo, Wairimu Chege, Tobias F. Rinke de Wit, Eduard J. Sanders, Susan M. Graham

**Affiliations:** 1 Kenya Medical Research Institute-Wellcome Trust Research Programme, Kilifi, Kenya; 2 Prevention Sciences Program, Division of AIDS (DAIDS), National Institute of Allergy and Infectious Diseases (NIAID), National Institutes of Health (NIH), Rockville, MD USA; 3 Department of Global Health, Amsterdam Institute for Global Health and Development, Academic Medical Centre, University of Amsterdam, Amsterdam, the Netherlands; 4 Nuffield Department of Medicine, Centre for Tropical Medicine and Global Health, University of Oxford, Oxford, UK; 5 Departments of Global Health, Medicine, and Epidemiology, University of Washington, Seattle, USA

**Keywords:** HIV testing, Coverage, Primary care, Acute HIV

## Abstract

**Background:**

HIV testing is the first step to stop transmission. We aimed to evaluate HIV testing history and new diagnoses among adult outpatients in Kenya aged 18–39 years seeking care for symptoms of acute HIV infection (AHI).

**Methods:**

The *Tambua Mapema Plus* study, a stepped-wedge trial, enrolled patients presenting to care at six primary care facilities with symptoms of AHI for a targeted HIV-1 nucleic acid (NA) testing intervention compared with standard provider-initiated testing using rapid antibody tests. Intervention participants underwent a questionnaire and NA testing, followed by rapid tests if NA-positive. Multinomial logistic regression was used to analyse factors associated with never testing or testing > 1 year ago (“late retesting”) relative to testing ≤ 1 year ago (“on-time testers”). Logistic regression was used to analyse factors associated with new diagnosis. All analyses were stratified by sex.

**Results:**

Of 1,500 intervention participants, 613 (40.9%) were men. Overall, 250 (40.8%) men vs. 364 (41.0%) women were late retesters, and 103 (16.8%) men vs. 50 (5.6%) women had never tested prior to enrolment. Younger age, single status, lower education level, no formal employment, childlessness, sexual activity in the past 6 weeks, and > 1 sexual partner were associated with testing history among both men and women. Intimate partner violence > 1 month ago, a regular sexual partner, and concurrency were associated with testing history among women only. New diagnoses were made in 37 (2.5%) participants (17 men and 20 women), of whom 8 (21.6%) had never tested and 16 (43.2%) were late retesters. Newly-diagnosed men were more likely to have symptoms for > 14 days, lower education level and no religious affiliation and less likely to be young, single, and childless than HIV-negative men; newly-diagnosed women were more likely to report fever than HIV-negative women. Among men, never testing was associated with fivefold increased odds (95% confidence interval 1.4–20.9) of new diagnosis relative to on-time testers in adjusted analyses.

**Conclusion:**

Most new HIV diagnoses were among participants who had never tested or tested > 1 year ago. Strengthening provider-initiated testing targeting never testers and late retesters could decrease time to diagnosis among symptomatic adults in coastal Kenya.

**Trial registration:**

ClinicalTrials.gov Identifier: NCT03508908 registered on 26/04/2018.

## Background

Globally, an estimated one in five people living with HIV (PLWH) are unaware of their HIV status [1]. Within Eastern and Southern Africa, groups that have gaps in terms of access to and uptake of HIV testing services (HTS) include key populations, partners of PLWH, men and young people [1]. The UNAIDS 2020 report highlights sex disparities in the HIV epidemic, with new infections decreasing more rapidly among women than among men and boys globally [2]. In Eastern and Southern Africa, despite a larger burden of women infected with HIV compared to men, testing rates, treatment coverage and viral suppression were higher among women, resulting in lower AIDS-related mortality rates among women compared to men in 2019 [2].

Several factors are associated with a lower likelihood of HIV testing in studies conducted in sub-Saharan Africa (SSA) including younger age (adolescents), male sex, less than primary education, lower socioeconomic status and having multiple sexual partners [3]. Masculine norms influence men’s engagement in the HIV care continuum in SSA, including HIV testing [4], clinic attendance, ART initiation and treatment continuation [5, 6]. Additional problems contributing to low HIV test uptake among men include stigma, discrimination and low risk perception, often leading to diagnosis with advanced disease and delays in care linkage [4, 5, 7–13]. Amongst women, HIV testing has largely been integrated within antenatal care (ANC) services and has led to greater test coverage for women, with an estimated 60.7% of women in SSA receiving HIV testing as part of ANC [14].

Kenya has an estimated 1.3 million PLWH, with a national HIV prevalence of 4.9% in 2018, higher among women than men (6.6% vs. 3.1%); in the same year, 36,000 new HIV infections were estimated [15]. An estimated 62% of all new infections were among young people aged 15–29 years, with 15–24-year-olds contributing 42% in 2020 [16]. Kenya’s HIV testing policies are geared towards attainment of the 2020 UNAIDS first 90 goal (i.e., that 90% of PLWH be aware of their status): in 2018, 79.5% of Kenyan PLWH were aware of their status, 82.7% among women and 72.6% among men [15]. By 2019, an estimated 90% of PLWH knew their status [17]. With the new UNAIDS goals to diagnose 95% of all PLWH by 2030, additional interventions are needed [18]. Gender-targeted interventions are critical to address the sex disparities in the HIV epidemic.

Kenyan national HTS guidelines recommend annual HIV testing for the general population and quarterly testing amongst those at increased risk of HIV acquisition [19]. With provider initiated testing and counselling (PITC) accounting for the majority (77%) of more than 13 million tests performed in Kenya in 2017–2018 [20], health facilities are an important site for intensified HIV testing. Using data obtained from the intervention phase of a stepped-wedge trial evaluating opt-out HIV-1 nucleic acid (NA) testing among young adult patients seeking care who met criteria for acute HIV infection (AHI) risk [21], we assessed HIV testing history and new HIV diagnoses among male and female patients aged 18–39 years presenting at six outpatient clinics, evaluating factors associated with each outcome separately for men and for women.

## Methods

### Study design

The *Tambua Mapema Plus* (TMP) study (ClinicalTrials.gov Identifier: NCT03508908) was a proof-of-concept trial assessing the impact of a health facility-based HIV testing intervention for detection of acute and chronic HIV infections using a point-of-care (POC) HIV-1 NA assay (Cepheid GeneXpert® HIV-1 Qual) compared with standard care among adult patients seeking care who met criteria for AHI risk [21]. The study recruited patients presenting at four public and two private primary care facilities in Mombasa and Kilifi counties (estimated HIV prevalence in 2018, 5.6% and 2.3%, respectively) [15] along the Kenyan coast, where febrile illnesses are most commonly caused by bacterial infections and common respiratory viruses, but malaria, dengue, and chikungunya infections are also endemic [22–26]. The trial used a modified stepped-wedge design to evaluate the yield of the HIV-1 RNA testing intervention at the six facilities among 1,500 participants in the intervention phase, after an observation period in which 1,375 participants were recruited [20]. The present analysis includes data collected during the intervention period of the study.

### Study setting and population

The six study facilities were chosen based on their proximity (within a 20-km radius) to a Kenya Medical Research Institute (KEMRI) research clinic north of Mombasa, an outpatient volume ≥ 15 outpatient visits daily, and the availability of HIV testing services. Facilities chosen included 1 public dispensary, 3 health centres (2 public, 1 private) and the outpatient departments of 2 hospitals (1 public, 1 private). Eligibility criteria for participation included: 1) age 18–39 years; 2) not previously diagnosed with HIV infection; and 3) an AHI risk score ≥ 2, with 1 point each for age 18–29 years, fever, fatigue, body pains, diarrhoea, or sore throat and 3 points for genital ulcer disease. This risk score was developed in our prior work, in which participants aged 18–29 and those with the specific symptoms and findings included were predictive of AHI [27, 28]. Up to 20 participants were targeted for enrolment each week (maximum of 4 per day) during facility working hours. Research staff were present at the facility over a range of time periods i.e. daytime, evening and weekends to ensure recruitment targets were met. Participants not meeting the inclusion criteria or who were unwilling or unable to participate due to time constraints or illness were excluded [21].

### Data collection

Data were collected from December 2017 to March 2020. Research staff (clinician or counsellors) obtained permission from the facility providers and study participants to be present during patient consultations and collected data on age, symptoms, axillary temperature, HIV testing history and HIV status for eligibility screening. Eligible patients who enrolled in the study were administered a computer-assisted self-interview (CASI) questionnaire collecting data on sociodemographic factors, sexual activity in the past 6 weeks, and if sexually active, details on sexual partners, condom use, and concurrency amongst the last three sexual partners. After completing the CASI, participants were tested first with the POC HIV-1 Qual assay, then tested with rapid HIV tests if positive, to distinguish acute from chronic HIV infection. All newly diagnosed participants were offered immediate linkage to HIV care and treatment and assisted partner services at a KEMRI research clinic. Detailed study procedures have been published [21].

### Data analysis

Descriptive statistics were used to summarize frequencies and percentages for categorical data and medians and interquartile ranges for continuous data. Comparisons by sex were made using Wilcoxon rank sum tests for continuous variables and Chi-square or Fisher’s exact tests for categorical variables, as appropriate.

Multinomial logistic regression was used to analyse sociodemographic and sexual risk factors associated with never testing or testing > 1 year ago (“late retesting”) relative to a reference category of last testing for HIV ≤ 1 year ago (“on-time testing”). Binomial logistic regression was used to analyse sociodemographic and sexual risk factors associated with a new HIV diagnosis, relative to testing negative. Crude relative risk ratios (RRR) and odds ratios (ORs) were calculated for factors associated with testing history and new HIV diagnosis, respectively. All analyses were stratified by sex. No multivariable analysis was conducted, as there was no pre-specified hypothesis.

In a separate analysis, the association between testing history and new HIV diagnosis was analysed overall and by sex, with and without adjustment for those factors associated with both testing history and new HIV diagnosis at *p* < 0.10 in bivariable analysis. Associations with a p value of ≤ 0.05 in multivariable modelling were considered significant. Data cleaning and analysis were conducted using Stata 15 (StataCorp, USA).

## Results

### Characteristics of study participants

**Fig. 1:**
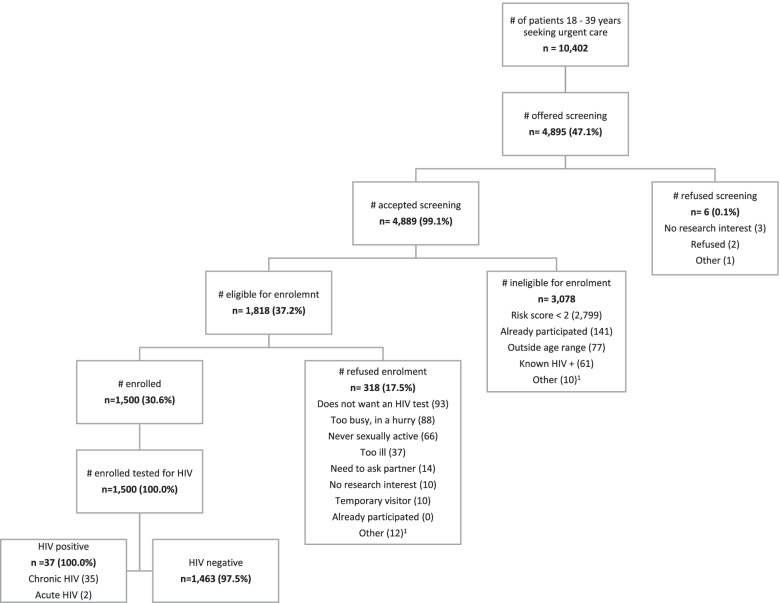
Study recruitment and outcomes for the intervention phase, Tambua Mapema Plus trial

Figure 1 presents a flow diagram of study recruitment and testing outcomes. In total, 613 men and 887 women enrolled between December 2017 and March 2020. Table 1 presents characteristics of the 1,500 participants. Men were older than women (median [interquartile range] age, 26.4 [23.3–30.8] years vs 25.1 [22.2–28.8] years; *p* < 0.001). Most participants had been sexually active in the past 6 weeks. Women were more likely to have experienced intimate partner violence (IPV) and less likely to be sexually active than men. Compared to women, more men were single, had secondary education or higher, were employed, and were childless. Men were more likely to report fever or have a temperature ≥ 37.5° Celsius, whereas women were more likely to report symptoms starting ≤ 14 days ago.

**Table 1:** Clinical, sociodemographic, and sexual behaviour characteristics of 1,500 study participants enrolled between December 2017 and March 2020

**Characteristics**	**Male** **N (%)**	**Female** **N (%)**	***P*** **value**
**Age**			
**30–39 years**	179 (29.2)	192 (21.7)	0.001
**25–29 years**	194 (31.7)	260 (29.3)	
**18–24 years**	240 (39.2)	435 (49.0)	
**Marital status**^**a**^			
**Married**	256 (41.8)	503 (56.7)	< 0.001
**Single**	334 (54.5)	319 (36.0)	
**Separated, Divorced, Widowed**	22 (3.6)	65 (7.3)	
**Level of education**^**a**^			
**Higher education**	197 (32.1)	187 (21.1)	< 0.001
**Secondary**	223 (36.4)	303 (34.2)	
**Primary and below**	192 (31.3)	397 (44.8)	
**Religion**^**a**^			
**Christian**	466 (76.0)	697 (78.6)	0.123
**Muslim**	132 (21.5)	181 (20.4)	
**None**	14 (2.9)	9 (1.0)	
**Source of income**^**a**^			
**Employed**	391 (63.8)	431 (48.6)	< 0.001
**Unemployed**	105 (17.1)	354 (39.9)	
**Casual labourers**	116 (18.9)	102 (11.5)	
**Having children**^**a**^			
**Yes**	261 (42.6)	585 (66.0)	< 0.001
**No**	351 (57.3)	302 (34.1)	
**Time since last sexual activity**^**a**^			
** ≤ 6 weeks ago**	414 (67.5)	679 (76.6)	0.001
**More than 6 weeks ago**	191 (31.2)	203 (22.9)	
**Never had sex**	7 (1.1)	5 (0.6)	
**Experience of intimate partner violence (IPV)**^**b**^			
**Never**	506 (82.5)	673 (75.9)	0.007
**Any IPV in the past 1 month**	25 (4.1)	41 (4.6)	
**Any IPV > 1 month ago**	81 (13.2)	173 (19.5)	
**Temperature**			
** < 37.5**	499 (81.4)	764 (86.1)	0.014
** ≥ 37.5**	114 (18.6)	123 (13.9)	
**Reported fever**	324 (52.9)	396 (44.6)	0.002
**Reported fatigue**	473 (77.2)	695 (78.4)	0.584
**Reported body aches**	410 (66.9)	603 (68.0)	0.655
**Reported diarrhoea**	104 (17.0)	138 (15.6)	0.466
**Reported sore throat**	193 (31.5)	255 (28.8)	0.255
**Reported genital ulcers**	42 (6.9)	71 (8.0)	0.406
**Days since symptoms began**			
** ≤ 14 days**	569 (92.8)	849 (95.7)	0.015
**Time since last HIV test**			
** ≤ 1 year ago**	260 (42.4)	473 (53.3)	< 0.001
**More than 1 year ago**	250 (40.8)	364 (41.0)	
**Never tested**	103 (16.8)	50 (5.6)	
***Sexual behaviour among 414 male and 679 female participants sexually active in the past 6 weeks***^***c***^			
**Risk group**			
**Sexually active general population**	396 (95.7)	655 (96.5)	0.497
**Sexually active key populations**^**’d**^	18 (4.4)	24 (3.5)	
**Self-reported number of sexual partners in the past 6 weeks**			
**1 partner**	321 (77.5)	625 (92.1)	< 0.001
** > 1 partner**	93 (22.5)	54 (8.0)	
**Nature of relationship with the most recent sexual partner in the past 6 weeks**			
**Spouse**	219 (52.9)	473 (69.7)	< 0.001
**Regular partner**^**e**^	93 (22.5)	125 (18.4)	
**Casual partner**	65 (15.7)	71 (10.5)	
**One-time encounter**	37 (8.9)	10 (1.5)	
**Condom use with the last sexual partner**^**f**^			
**No**	332 (80.2)	615 (90.6)	< 0.001
**Yes**	82 (19.8)	64 (9.4)	
**Age of last sexual partner in the past 6 weeks**			
**About the same age**	226 (54.6)	309 (45.5)	< 0.001
**More than 5 years older**	21 (5.1)	361 (53.2)	
**More than 5 years younger**	167 (40.3)	9 (1.3)	
**HIV status of last sexual partner in the past 6 weeks**			
**Partner status negative or unknown**	400 (96.6)	667 (98.2)	0.089
**Partner status positive**	14 (3.4)	12 (1.8)	
**Concurrency amongst the last three reported sexual partners**			
**No**	352 (85.0)	642 (94.6)	< 0.001
**Yes**	62 (15.0)	37 (5.5)	

^a^Data missing for 1 participant due to corrupted Computer-Assisted Self-Interview (CASI) software files

^b^Includes any experience of physical, emotional or sexual IPV

^c^Sexual risk behaviour data was only collected from participants who reported sexual encounters in the past 6 weeks

^d^Key populations included sex workers *n* = 37, men who have sex with men (MSM) *n* = 4 and people who inject drugs (PWID) *n* = 1

^e^Regular partner: long-term partner other than spouse

^f^Those who could not recall using a condom at their last sexual encounter (*n* = 6, 3 males and 3 females) were included in the no condom use category

Among participants sexually active in the past 6 weeks (414 men and 679 women), most reported no same-sex behaviour or transactional sex (Table 1). Compared to men, women were more likely to report only one sexual partner, a spouse as their most recent sexual partner, no condom use with their most recent sexual partner, and HIV-negative or unknown status of their most recent sexual partner. Based on reported times of first and last sexual encounters with their last three sexual partners, most participants (90.9%) had no concurrent partners. While most men reported being the same age as their last sexual partner, most women reported that their last sexual partner was > 5 years older.

### HIV testing history

Among the 1,500 participants, 733 (48.9%) had tested in the past year, 614 (40.9%) had tested more than a year ago, and 153 (10.2%) had never tested. Testing differed by sex, with 260 men (42.4%) testing in the past year, 250 (40.8%) testing late, and 103 (16.8%) never having tested, compared to 473 women (53.3%) testing in the past year, 364 (41.0%) testing late, and 50 (5.6%) never having tested prior to study enrolment (Chi square *p* < 0.001) (Table 1).

### HIV testing among men

Table 2 presents factors associated with late retesting and never testing among men. Lower education level (relative to higher education), casual labor (relative to formal employment) and reporting no sex in the past 6 weeks (relative to reporting sex in the past 6 weeks) were associated with a higher likelihood of late retesting. No sexual behaviours were associated with late retesting among sexually active men. Younger age (18–24 years) (relative to 30–39 years), single marital status (relative to married), lower education level, childlessness (relative to having children) and reporting no sex in the past 6 weeks were associated with a higher likelihood of never testing. Among sexually active men, having more than one sexual partner in the past 6 weeks (relative to one partner) was associated with a lower likelihood of never testing for HIV. No clinical symptoms were associated with testing history.

**Table 2:** Factors associated with HIV testing history among 613 male and 887 female symptomatic outpatients

**Multinomial logistic regression of HIV testing history outcome, with on-time testing (i.e., testing ≤ 1 year) as the reference category**								
	**Males**				**Females**			
	**Tested > 1 year ago**		**Never tested**		**Tested > 1 year ago**		**Never tested**	
**Participant characteristics**	**RRR (95% CI)**	**Wald** ***p*** **value**	**RRR (95% CI)**	**Wald** *p* **value**	**RRR (95% CI)**	**Wald** *p* **value**	**RRR (95% CI)**	**Wald** *p* **value**
**Age**								
**30–39 years**	[ref]		[ref]		[ref]		[ref]	
**25–29 years**	0.81 (0.52, 1.25)	0.341	0.90 (0.47, 1.74)	0.766	0.89 (0.61, 1.30)	0.540	6.5 (0.81, 52.10)	0.078
**18–24 years**	0.74 (0.48, 1.13)	0.163	1.91 (1.07, 3.40)	0.028	0.90 (0.64, 1.28)	0.566	18.49 (2.51, 136.33)	0.004
**Marital status**^**a**^								
**Married**	[ref]		[ref]		[ref]		[ref]	
**Single**	1.01 (0.71, 1.43)	0.977	1.93 (1.18, 3.17)	0.009	1.06 (0.79, 1.43)	0.689	8.31 (3.93, 17.55)	< 0.001
**Separated, Divorced, Widowed**	0.83 (0.32, 2.18)	0.703	1.53 (0.45, 5.23)	0.495	0.94 (0.55, 1.58)	0.809	-	-
**Level of education**^**a**^								
**Higher education**	[ref]		[ref]		[ref]		[ref]	
**Secondary**	1.84 (1.21,2.80)	0.004	2.02 (1.11, 3.65)	0.021	1.08 (0.73, 1.59)	0.700	0.95 (0.48, 1.87)	0.881
**Primary and below**	1.57 (1.01, 2.44)	0.046	2.60 (1.44, 4.69)	0.001	1.13 (0.78, 1.62)	0.521	0.29 (0.13, 0.67)	0.003
**Religion**^**a**^								
**Christian**	[ref]		[ref]		[ref]		[ref]	
**Muslim**	1.53 (0.99, 2.34)	0.054	1.33 (0.76, 2.34)	0.321	0.89 (0.63, 1.24)	0.483	0.39 (0.15, 1.01)	0.053
**None**	0.37 (0.10, 1.39)	0.142	0.59 (0.13, 2.80)	0.508	2.56 (0.63, 10.32)	0.187	-	-
**Source of income**^**a**^								
**Employed**	[ref]		[ref]		[ref]		[ref]	
**Unemployed**	0.94 (0.58, 1.53)	0.799	1.53 (0.86, 2.75)	0.150	0.93 (0.69, 1.24)	0.616	1.87 (1.02, 3.45)	0.044
**Casual labourers**	1.74 (1.10, 2.77)	0.019	1.58 (0.85, 2.92)	0.146	1.19 (0.77, 1.84)	0.437	0.47 (0.11, 2.08)	0.319
**Having children**^**a**^								
**Yes**	[ref]		[ref]		[ref]		[ref]	
**No**	0.92 (0.65, 1.30)	0.627	1.54 (0.95, 2.48)	0.079	1.19 (0.88, 1.60)	0.254	59.47 (14.25, 248.11)	< 0.001
**Time since last sexual activity**^**a**^								
** ≤ 6 weeks ago**	[ref]		[ref]		[ref]		[ref]	
**More than 6 weeks ago**	1.59 (1.08, 2.34)	0.018	2.20 (1.35, 3.57)	0.002	1.44 (1.03, 2.02)	0.031	5.18 (2.84, 9.47)	< 0.001
**Never had sex**	1.80 (0.30, 10.88)	0.524	3.34 (0.46, 24.27)	0.232	2.12 (0.35, 12.76)	0.413	-	-
**Experience of IPV**^**a, b**^								
**Never**	[ref]		[ref]		[ref]		[ref]	
**Any IPV in the past 1 month**	1.74 (0.71, 4.28)	0.230	1.23 (0.36, 4.18)	0.743	0.77 (0.40, 1.46)	0.419	-	-
**Any IPV > 1 month ago**	1.04 (0.63, 1.72)	0.880	0.68 (0.32, 1.43)	0.312	0.70 (0.50, 1.00)	0.049	0.20 (0.06, 0.67)	0.009
**Temperature**								
** < 37.5**	[ref]		[ref]		[ref]		[ref]	
** ≥ 37.5**	1.17 (0.74, 1.84)	0.503	1.53 (0.87, 2.69)	0.136	1.20 (0.81, 1.78)	0.359	1.10 (0.47, 2.55)	0.825
**Reported fever**	1.23 (0.87, 1.75)	0.240	1.19 (0.75, 1.88)	0.453	0.98 (0.74, 1.29)	0.884	1.61 (0.89, 2.89)	0.113
**Reported fatigue**	0.99 (0.66, 1.49)	0.971	1.27 (0.72, 2.24)	0.405	1.12 (0.80, 1.57)	0.497	0.66 (0.35, 1.25)	0.201
**Reported body aches**	0.93 (0.64, 1.34)	0.683	1.08 (0.66, 1.76)	0.765	1.31 (0.97, 1.77)	0.073	0.65 (0.36, 1.17)	0.153
**Reported diarrhoea**	0.78 (0.49, 1.23)	0.281	0.72 (0.38, 1.34)	0.297	0.88 (0.60, 1.28)	0.496	0.98 (0.44, 2.17)	0.959
**Reported sore throat**	1.04 (0.72, 1.51)	0.830	0.71 (0.42, 1.18)	0.188	0.95 (0.70, 1.29)	0.746	0.68 (0.33, 1.35)	0.262
**Reported genital ulcers**	0.78 (0.40, 1.53)	0.466	0.58 (0.21, 1.58)	0.288	0.79 (0.47, 1.31)	0.363	0.65 (0.20, 2.20)	0.493
**Days since symptoms began**								
** ≤ 14 days**	0.59 (0.29, 1.18)	0.136	0.68 (0.27, 1.66)	0.394	0.72 (0.37, 1.39)	0.326	1.94 (0.25, 14.83)	0.524
***Sexual risk behaviour characteristics among 414 male and 679 female participants sexually active in the past 6 weeks***								
**Risk group**								
**Sexually active general population**	[ref]	0.731	[ref]	0.290	[ref]		[ref]	
**Sexually active key population**^**c**^	1.21 (0.41, 3.51)		1.98 (0.56, 7.01)		0.75 (0.31, 1.78)	0.509	1.12 (0.14, 8.86)	0.916
**Self-reported number of sexual partners in the past 6 weeks**								
**1 partner**	[ref]		[ref]		[ref]		[ref]	
** > 1 partner**	1.09 (0.67, 1.77)	0.727	0.37 (0.15, 0.92)	0.032	0.40 (0.20, 0.77)	0.006	0.82 (0.19, 3.62)	0.792
**Nature of relationship with the most recent sexual partner in the past 6 weeks**								
**Spouse**	[ref]		[ref]		[ref]		[ref]	
**Regular partner**^**d**^	0.69 (0.41, 1.17)	0.167	0.90 (0.42, 1.92)	0.781	0.85 (0.56, 1.29)	0.448	2.97 (1.13, 7.79)	0.027
**Casual partner**	1.03 (0.56, 1.89)	0.922	1.47 (0.65, 3.33)	0.355	0.98 (0.58, 1.64)	0.933	2.74 (0.82, 9.16)	0.102
**One-time encounter**	1.04 (0.47, 2.26)	0.930	2.00 (0.77, 5.20)	0.157	0.68 (0.17, 2.76)	0.590	4.45 (0.49, 40.53)	0.185
**Condom use with the last sexual partner **^**e**^								
**No**	[ref]		[ref]		[ref]		[ref]	
**Yes**	0.68 (0.40, 1.16)	0.159	0.80 (0.38, 1.67)	0.548	0.76 (0.44, 1.33)	0.339	2.53 (0.89, 7.20)	0.082
**Age of last sexual partner in the past 6 weeks**								
**About the same age**	[ref]		[ref]		[ref]		[ref]	
**More than 5 years older**	2.18 (0.81, 5.87)	0.122	1.63 (0.40, 6.68)	0.498	1.11 (0.81, 1.52)	0.506	0.68 (0.29, 1.58)	0.369
**More than 5 years younger**	1.31 (0.85, 2.03)	0.218	1.30 (0.71, 2.39)	0.395	1.88 (0.50, 7.16)	0.352	-	-
**HIV status of last sexual partner in the past 6 weeks**								
**Partner status negative or unknown**	[ref]	0.605	[ref]	0.403	[ref]	0.547	[ref]	-
**Partner status positive**	0.74 (0.24, 2.31)		0.41 (0.05, 3.33)		1.42 (0.45, 4.45)		-	
**Concurrency amongst the last three reported sexual partners**								
**No**	[ref]		[ref]		[ref]		[ref]	
**Yes**	1.46 (0.83, 2.57)	0.186	0.46 (0.15, 1.37)	0.162	0.45 (0.21, 0.98)	0.044	0.60 (0.08, 4.63)	0.625

*Abbreviations*: *IPV* intimate partner violence, *ref* reference category, *RRR* relative-risk ratio

^a^Data missing for 1 participant due to corrupted Computer-Assisted Self-Interview software files

^b^Includes any experience of physical, emotional and sexual IPV

^c^Key populations included sex workers (*n* = 13), men who have sex with men (*n* = 4) and people who inject drugs (*n* = 1)

^d^Regular partner: long-term partner other than spouse

^e^Those who could not recall using a condom at their last sexual encounter (*n* = 6, 3 males, 3 females) were included in the no condom use category

### HIV testing among women

Table 2 also presents factors associated with late retesting and never testing among women. Reporting no sex in the past 6 weeks was associated with a higher likelihood, while having experienced IPV over a month ago (relative to never having experienced IPV) was associated with a lower likelihood of late retesting. Among sexually active women, reporting multiple sexual partners in the past 6 weeks and reporting concurrency among the last three sexual partners (relative to no concurrency) were associated with a lower likelihood of late retesting. Younger age (18–24 years), single marital status, unemployment, childlessness and reporting no sex in the past 6 weeks were associated with a higher likelihood of never testing. Primary and below education level and having experienced IPV over a month ago were associated with a lower likelihood of never testing. Among sexually active women, those who reported that their last sexual encounter was with a regular partner were almost 3 times as likely to have never tested for HIV, compared to those who reported that their last sexual encounter was with a spouse. No clinical symptoms were associated with testing history.

### New HIV diagnosis

Using POC HIV-1 Qual testing, 37 (2.5%) participants were newly diagnosed with HIV infection: 17 men (2.8%) and 20 women (2.3%, *p* = 0.52). Among men, all new diagnoses were chronic HIV; among women, there were 18 (2.0%) chronic and 2 (0.2%) acute cases. Six (35.3%) of 17 newly diagnosed men vs. 15 (75%) of 20 newly diagnosed women were aged 18–29 years (Chi square *p* = 0.02).

### Factors associated with new HIV diagnosis

Among men, factors associated with a lower odds of new HIV diagnosis included younger age, single marital status, childlessness and symptoms that started ≤ 14 days ago (relative to > 14 days ago). Lower education level and no religious affiliation (relative to Christianity) were associated with an increased odds of new HIV diagnosis among men, and being separated, widowed, or divorced had a borderline association with this outcome. Recent sexual behaviours were not associated with a new HIV diagnosis among men (Table 3). Among women, reported fever was associated with increased odds of a new HIV diagnosis. No sociodemographic factors or sexual behaviours were associated with new HIV diagnosis among women (Table 3).

**Table 3:** Factors associated with new HIV diagnosis among 613 male and 887 female symptomatic outpatients with HIV-negative or unknown status

**Logistic regression for new HIV diagnosis outcome**				
**Participant characteristics**	**Males**		**Females**	
	**OR (95% CI)**	**Wald p value**	**OR (95% CI)**	**Wald p value**
**Age**				
**30–39 years**	[ref]		[ref]	
**25–29 years**	0.16 (0.03, 0.73)	0.018	1.50 (0.50, 4.45)	0.469
**18–24 years**	0.26 (0.08, 0.83)	0.023	0.44 (0.12, 1.52)	0.192
**Marital status**^**a**^				
**Married**	[ref]		[ref]	
**Single**	0.30 (0.09, 0.96)	0.043	1.11 (0.42, 2.94)	0.840
**Separated, Divorced, Widowed**	3.88 (0.99, 15.32)	0.053	2.39 (0.64, 8.90)	0.196
**Level of education**^**a**^				
**Higher education**	[ref]		[ref]	
**Secondary**	0.88 (0.18, 4.42)	0.878	1.24 (0.22, 6.82)	0.807
**Primary and below**	3.93 (1.08, 14.31)	0.038	3.38 (0.76, 15.03)	0.110
**Religion**^**a**^				
**Christian**	[ref]		[ref]	
**Muslim**	1.29 (0.40, 4.13)	0.665	0.67 (0.20, 2.33)	0.533
**None**	6.89 (1.38, 34.56)	0.019	-	-
**Source of income**^**a**^				
**Employed**	[ref]		[ref]	
**Unemployed**	0.82 (0.18, 3.87)	0.807	0.97 (0.38, 2.49)	0.955
**Casual labourers**	2.32 (0.81, 6.65)	0.119	0.84 (0.18, 3.90)	0.826
**Having children**^**a**^				
**Yes**	[ref]		[ref]	
**No**	0.04 (0.01, 0.33)	0.002	0.48 (0.16, 1.44)	0.189
**Time since last sexual activity**^**a**^				
** ≤ 6 weeks ago**	[ref]		[ref]	
**More than 6 weeks ago**	0.90 0.31, 2.59)	0.846	0.83 (0.28, 2.52)	0.746
**Never had sex**	-	-	-	-
**Experience of IPV**^**a,b**^				
**Never**	[ref]		[ref]	
**Any IPV in the past 1 month**	1.46 (0.18, 11.60)	0.718	-	-
**Any IPV > 1 month ago**	0.89 (0.20, 3.99)	0.879	1.69 (0.64, 4.47)	0.289
**Temperature**				
** < 37.5**	[ref]		[ref]	
** ≥ 37.5**	1.36 (0.43, 4.25)	0.597	2.12 (0.75, 5.93)	0.154
**Reported fever**	1.00 (0.38, 2.64)	0.994	2.96 (1.13, 7.78)	0.028
**Reported fatigue**	1.39 (0.39, 4.92)	0.607	0.50 (0.20, 1.28)	0.150
**Reported body aches**	0.55 (0.21, 1.44)	0.222	0.46 (0.19, 1.12)	0.088
**Reported diarrhoea**	1.53 (0.49, 4.78)	0.468	0.96 (0.28, 3.31)	0.944
**Reported sore throat**	1.54 (0.58, 4.12)	0.386	1.67 (0.68, 4.14)	0.266
**Reported genital ulcers**	1.85 (0.41, 8.39)	0.423	1.29 (0.29, 5.65)	0.740
**Days since symptoms began**				
** ≤ 14 days**	0.12 (0.04, 0.36)	< 0.001	0.39 (0.09, 1.74)	0.218
**Time since last HIV test**				
** ≤ 1 year ago**	[ref]		[ref]	
**More than 1 year ago**	1.04 (0.30, 3.64)	0.950	1.81 (0.72, 4.55)	0.206
**Never tested**	3.72 (1.15, 12.00)	0.028	1.19 (0.15, 9.68)	0.873
***Sexual risk behaviour characteristics among 414 male and 679 female participants sexually active in the past 6 weeks***				
**Risk group**				
**Sexually active general population**	[ref]	0.054	[ref])	
**Sexually active key population**^**c**^	4.83 (0.98, 23.86)		-	-
**Self-reported number of sexual partners in the past 6 weeks**				
**1 partner**	[ref]		[ref]	
** > 1 partner**	2.55 (0.79, 8.23)	0.118	-	-
**Nature of relationship with the most recent sexual partner in the past 6 weeks**				
**Spouse**	[ref]		[ref]	
**Regular partner**^**d**^	0.39 (0.05, 3.25)	0.381	1.93 (0.65, 5.75)	0.238
**Casual partner**	1.72 (0.42, 7.07)	0.453	0.66 (0.08, 5.25)	0.696
**One-time encounter**	2.03 (0.39, 10.45)	0.398	-	-
**Condom use with the last sexual partner**^**e**^				
**No**	[ref]		[ref]	
**Yes**	3.01 (0.93, 9.75)	0.065	2.28 (0.63, 8.21)	0.209
**Age of last sexual partner in the past 6 weeks**				
**About the same age**	[ref]		[ref]	
**More than 5 years older**	2.21 (0.25, 19.85)	0.479	1.10 (0.41, 3.00)	0.847
**More than 5 years younger**	1.65 (0.49, 5.49)	0.417	-	-
**HIV status of last sexual partner in the past 6 weeks**				
**Partner status negative or unknown**	[ref]		[ref]	0.202
**Partner status positive**	-	-	3.95 (0.48, 32.60)	
**Concurrency amongst the last three reported sexual partners**				
**No**	[ref]		[ref]	
**Yes**	1.94 (0.51, 7.37)	0.332	-	-

*Abbreviations*: *IPV* intimate partner violence, *OR* odds ratio, *ref* reference category

^a^Data missing for 1 male participant due to corrupted Computer-Assisted Self-Interview software files

^b^Includes any experience of physical, emotional and sexual IPV

^c^Key populations included, for males: sex workers (*n* = 13), men who have sex with men (*n* = 4) and people who inject drugs (*n* = 1), and for females: sex workers (*n* = 24)

^d^Regular partner: long-term partner other than spouse

^e^Those who could not recall using a condom at their last sexual encounter (*n* = 6, 3 males and 3 females) were included in the no condom use category

### New HIV diagnosis and testing history

Overall, 13 (35.1%) of new diagnoses were among on-time retesters, 16 (43.2%) among late retesters, and 8 (21.6%) among those who had never tested for HIV (Chi square *p* = 0.041). (Table 4). New HIV diagnoses were made in 5 (29.4%) who had tested in the past year, 5 (29.4%) who were late retesters and 7 (41.2%) men who were never testers, compared to 8 (40.0%) who had tested in the last year, 11 (55.0%) who were late retesters and 1 (5.0%) who had never tested. In unadjusted analyses (Table 3), never testing was associated with a 3.7-fold increased odds of a new HIV diagnosis relative to on-time testing in men. After adjustment for factors associated with both testing history and a new HIV diagnosis, never testing was associated with fivefold increased odds (adjusted odds ratio 5.4, 95% CI 1.4–20.9) of a new HIV diagnosis among men. There was no association between testing history and new HIV diagnosis among women in adjusted or unadjusted analysis.

**Table 4:** Association between HIV testing history and new HIV diagnosis among 1500 symptomatic outpatients

	**New diagnoses** **(n, % of diagnoses)**	**Odds Ratio** **(95% Confidence Interval)**	**Wald p value**	**Adjusted Odds Ratio (95% Confidence Interval)**	**Wald p value**
Testing history, among 613 male participants^a^					
Within past year	5 (29.4)	Reference		Reference	
> 1 year ago	5 (29.4)	1.04 (0.30, 3.64)	0.950	1.09 (0.28 4.22)	0.903
Never tested	7 (41.2)	3.72 (1.15, 12.00)	0.028	5.35 (1.37, 20.87)	0.016
Testing history, among 887 female participants^b^					
Within past year	8 (40.0)	Reference		Reference	
> 1 year ago	11 (55.0)	1.81 (0.72, 4.55)	0.206	1.91 (0.76, 4.83)	0.169
Never tested	1 (5.0)	1.19 (0.15, 9.68)	0.873	1.09 (0.13, 8.93)	0.937

^a^Adjusted for factors associated with both testing history and new HIV diagnosis at *p* < 0.10 including age, marital status, education level, religion and having children among males

^b^Adjusted for body aches, the only factor associated with both testing history and new HIV diagnosis at p < 0.10 among females

## Discussion

We evaluated factors associated with HIV test coverage and new HIV diagnosis among male and female adult outpatients aged 18–39 seeking care for symptoms of infectious illness at six health facilities in coastal Kenya. Our study highlights sex differences in the uptake of HIV testing, with more men than women reporting never having tested (16.8% vs 5.6%) and more women than men reporting on-time testing (53.3% vs 42.4%). Among both men and women, young, single individuals with no children were less likely to have tested. Associations between sexual behaviours (e.g., multiple partners, concurrency, IPV) and HIV testing were evident among women, but no associations between sexual behaviour and HIV testing were found among men. In contrast, never testing for HIV prior to the study was associated with a fivefold increased odds of a new HIV diagnosis relative to on-time testing among men. Among men, a new HIV diagnosis was associated with reporting symptoms that started > 14 days ago, while fever was associated with a new diagnosis among women, suggesting earlier care-seeking by women in this population. Of note, both AHI cases were diagnosed among women. These findings point to a need for greater engagement of both men and women in the study area in HIV testing and prevention measures.

Gender norms have been shown to influence engagement in the HIV care continuum [4, 5]. Men have traditionally viewed health facilities as feminine spaces, with fear of embarrassment and showing weakness when seen seeking treatment, which has often led to delayed linkage to care. These effects have been compounded by HIV-associated stigma and fear that HIV would compromise a man’s ability to work and their role as a provider or lead to an early death, affecting their willingness to test for HIV and engage in HIV prevention methods [4, 5, 7]. Interestingly in our study, men who reported more than one sexual partner in the past 6 weeks were more likely to have ever tested for HIV, reflecting engagement with HTS among those with risky sexual practices. Despite a relatively high proportion of men who had never tested before in our study, all enrolled participants (613 males) accepted HIV testing when presenting for health care due to symptoms. Indeed, in the observational phase of the present study, we demonstrated high acceptability of HIV testing among men (~ 93.0%) in our target population [29]; similar findings have been documented in other parts of SSA [8]. This suggests missed opportunities for facility-based testing of symptomatic men in our setting that could be addressed with targeted interventions.

Structural barriers contribute to low testing and linkage rates among men in Kenya and other SSA settings. These barriers include labour opportunities requiring extended absence from households, long wait times and inconvenient clinic hours incompatible with work schedules, leading some men to assume their HIV status by proxy, adopting their wives’ HIV status as their own [7, 9, 12]. In our study, men who reported casual labour were more likely to test late relative to those reporting full employment, suggesting constraints on their ability to test. While HIV testing outside of facilities (e.g., community, mobile HIV testing and counseling, home-based testing) is acceptable to men and can achieve higher test coverage [12, 30], facility-based PITC such as in the current study or facility-based self-testing as carried out in a recent study in Malawi [31] could decrease missed opportunities, inform clinical decision-making, and identify HIV-positive men earlier in the course of disease.

We found that women were more likely to be on-time testers and to have ever tested for HIV compared to men. However, despite high HIV test coverage among women in Kenya and other settings in SSA through ANC and sexual reproductive health services [7, 15, 30, 32, 33], young (18–24 years), single, unemployed women and those who were childless were more likely to have never tested in our study. This is of concern, as young women aged 15–24 years made up a third of new adult infections and 10% of total AIDS-related deaths in Kenya in 2017 [34]. Interventions to increase HTS uptake and prompt linkage to care or prevention services among youth are needed, even though testing yield (i.e., HIV test positivity) will be low in these groups. Studies in western Kenya demonstrated increased HTS coverage could be attained among adolescent girls and young women through mobile, home- or community-based and oral self-testing approaches [35].

We found that female participants engaging in riskier behaviours (i.e., multiple sexual partners, concurrency) had a lower likelihood of late retesting, suggesting a realistic assessment of their potential risk of HIV infection. Interestingly, we found that sexually active women whose last sexual encounter was with a regular partner were almost 3 times as likely to have never tested for HIV compared to those whose last sexual encounter was with a spouse. This could be due to a tendency to wait for marriage to test, or a fear of their partner’s negative reaction or rejection if women test positive [36]. Our data also showed that women who had ever experienced IPV (25% of total) were more likely to retest late. Experiences with violence can undermine condom negotiation, HIV testing and linkage to care if positive [36, 37]. Despite the integration of HTS in Gender Based Violence (GBV) services in Kenya [19], more efforts are needed to ensure engagement in the HIV care continuum among women experiencing IPV. Multi-pronged programmes aimed at increasing GBV awareness, addressing cultural norms and strengthening community responses have been beneficial in other SSA settings [38], and could be helpful for Kenyan women, along with greater access to education and employment opportunities that lead to women’s empowerment.

Our study enrolled symptomatic adults 18–39 years presenting for primary care to detect acute and chronic HIV infection, with most new diagnoses among late retesters occurring among women and a large number of diagnoses among young women aged 18–29. Both AHI cases were diagnosed in women, and women reporting fever as a symptom had increased odds of a new HIV diagnosis. This underscores the importance of HIV testing among patients, especially women, presenting to care with symptoms of AHI [27]. Among men, whose symptoms had often lasted longer than those of women, we only diagnosed chronic HIV infection. Our findings underscore the importance of prompt HIV testing among patients presenting with symptoms of AHI, which clearly provides benefits with respect to earlier HIV diagnosis and prompt linkage to care and treatment. In Kenya, PITC constitutes majority of HIV testing conducted yet remains incompletely implemented: during the observation phase of the *Tambua Mapema Plus* study, only 1 in 4 symptomatic patients 18–39 years seeking care were offered an HIV test (29). Scaling up PITC, addressing the barriers to its implementation, and increasing knowledge and awareness of retesting recommendations may further reduce the sex disparities in HIV test such as those documented in this study.

Our study has some limitations. First, we relied on self-reported HIV testing status and sexual behaviour, which may have been influenced by social desirability. Second, our study was conducted among adults aged 18–39 years seeking care at six health facilities in coastal Kenya who were at increased risk for of AHI based on a risk score algorithm. These results may not be generalizable to patients presenting for other reasons (e.g., trauma, hypertension), to symptomatic patients in other regions in Kenya, or to populations beyond our target age group. Third, we stratified the study population by sex, which reduced power but highlighted important differences between men and women. Despite these limitations, our study provides useful insights on the correlates of late retesting and never testing for HIV, and of new HIV diagnosis, among men and women seeking care for symptoms compatible with AHI or other acute infectious illnesses. Diagnostic evaluations in this population are important for clinical management, and as such, this population should be of high priority for HIV testing strategies.

## Conclusions

Our study evaluated the correlates of testing history and new HIV diagnosis among adults aged 18–39 years seeking care for symptoms of an acute infectious illness who met our risk score criteria. Our findings underscore the importance of facility-based HIV testing among adults presenting with symptoms of AHI, in order to avoid diagnostic delays and facilitate early linkage to HIV care and treatment. Our stratified analysis approach highlights the need for sex-targeted interventions. Approaches focused on scaling up provider-initiated HIV testing and improving HIV test uptake among key patient groups such as men who have never tested for HIV, and young, single men and women are needed. Opt-out HIV testing strategies that take into account HIV testing history (never testing or late retesting) could decrease time to diagnosis, greatly improve knowledge of HIV status and lead to better clinical outcomes. Addressing differences in HIV test uptake by men and women is key for achieving the UNAIDS 95–95-95 goals.

## Data Availability

The datasets generated and/or analysed during the current study will be made available in our institutional repository, KWTRP Dataverse: https://dataverse.harvard.edu/dataverse/kwtrp under managed access as per our institutional policies.

## Abbreviations

ANC: Antenatal Care services
ART: Anti-Retroviral Therapy
CASI: Computer Assisted Self-Interview
GBV: Gender Based Violence services
HTS: HIV Testing Services
IPV: Intimate Partner Violence
KEMRI: Kenya Medical Research Institute
MSM: Men who have sex with men
NA: Nucleic Acid
OR: Odds Ratio
PITC: Provider Initiated HIV Testing and Counselling
PLWH: People Living With HIV
POC: Point-Of-Care
PWID: People Who Inject Drugs
RRR: Relative Risk Ratios
SSA: Sub-Saharan Africa
UNAIDS: Joint United Nations Programme on HIV/AIDS

## References

[1] WHO. WHAT WORKS FOR GENERATING DEMAND FOR HIV TESTING SERVICES 2019 [Available from: https://www.who.int/publications/i/item/what-works-for-generating-demand-for-hiv-testing-services.

[2] UNAIDS. UNAIDS Data 2020 2020 [Available from: https://www.unaids.org/sites/default/files/media_asset/2020_aids-data-book_en.pdf.

[3] Staveteig S, Croft TN, Kampa KT, Head SK. Reaching the “first 90”: Gaps in coverage of HIV testing among people living with HIV in 16 African countries. PLoS One. 2017;12(10):e0186316-e. 10.1371/journal.pone.0186316 PMC5638499 29023510

[4] Sileo KM, Fielding-Miller R, Dworkin SL, Fleming PJ. What Role Do Masculine Norms Play in Men’s HIV Testing in Sub-Saharan Africa?: A Scoping Review. AIDS Behav. 2018;22(8):2468–79. 10.1007/s10461-018-2160-z PMC6459015 29777420

[5] Sileo KM, Fielding-Miller R, Dworkin SL, Fleming PJ (2019). A scoping review on the role of masculine norms in men’s engagement in the HIV care continuum in sub-Saharan Africa. AIDS Care.

[6] Ochieng-Ooko V, Ochieng D, Sidle JE, Holdsworth M, Wools-Kaloustian K, Siika AM (2010). Influence of gender on loss to follow-up in a large HIV treatment programme in western Kenya. Bull World Health Organ.

[7] DiCarlo AL, Mantell JE, Remien RH, Zerbe A, Morris D, Pitt B, et al. “Men usually say that HIV testing is for women”: gender dynamics and perceptions of HIV testing in Lesotho. Cult Health Sex. 2014;16(8):867–82. 10.1080/13691058.2014.913812 PMC4116609 24854495

[8] Quinn C, Kadengye DT, Johnson CC, Baggaley R, Dalal S (2019). Who are the missing men? Characterising men who never tested for HIV from population-based surveys in six sub-Saharan African countries. J Int AIDS Soc..

[9] Okal J, Lango D, Matheka J, Obare F, Ngunu-Gituathi C, Mugambi M, et al. “It is always better for a man to know his HIV status” - A qualitative study exploring the context, barriers and facilitators of HIV testing among men in Nairobi, Kenya. PLoS One. 2020;15(4):e0231645-e. 10.1371/journal.pone.0231645 PMC7159816 32294124

[10] Lakhe NA, Diallo Mbaye K, Sylla K, Ndour CT (2019). HIV screening in men and women in Senegal: coverage and associated factors; analysis of the 2017 demographic and health survey. BMC Infect Dis..

[11] Takarinda KC, Madyira LK, Mhangara M, Makaza V, Maphosa-Mutsaka M, Rusakaniko S (2016). Factors Associated with Ever Being HIV-Tested in Zimbabwe: An Extended Analysis of the Zimbabwe Demographic and Health Survey (2010–2011). PLoS One..

[12] Camlin CS, Ssemmondo E, Chamie G, El Ayadi AM, Kwarisiima D, Sang N, et al. Men “missing” from population-based HIV testing: insights from qualitative research. AIDS care. 2016;28(Suppl 3):67–73. 10.1080/09540121.2016.1164806 PMC5749410 27421053

[13] Peltzer K, Matseke G, Mzolo T, Majaja M (2009). Determinants of knowledge of HIV status in South Africa: results from a population-based HIV survey. BMC Public Health..

[14] Gunn JKL, Asaolu IO, Center KE, Gibson SJ, Wightman P, Ezeanolue EE (2016). Antenatal care and uptake of HIV testing among pregnant women in sub-Saharan Africa: a cross-sectional study. J Int AIDS Soc..

[15] NASCOP. KENPHIA 2018 Preliminary Report 2018 [Available from: https://www.nascop.or.ke/kenphia-report/.

[16] NACC/NASCOP. 2020 World AIDS Day Report: Kenya HIV Progress Indicators 2020 [Available from: https://app.box.com/s/kb1f0tkbjhlcn2a610too8vlpizxqla8.

[17] NACC. Kenya AIDS Strategic Framework II 2020/21 -2024/25 Sustain Gains, Bridge Gaps and Accelerate Progress [Available from: https://nacc.or.ke/wp-content/uploads/2021/01/KASFII_Web22.pdf.

[18] UNAIDS. UNDERSTANDING FAST-TRACK: ACCELERATING ACTION TO END THE AIDS EPIDEMIC BY 2030 2015 [Available from: https://www.unaids.org/sites/default/files/media_asset/201506_JC2743_Understanding_FastTrack_en.pdf.

[19] NASCOP. The Kenya HIV Testing Services Guidelines Ministry of Health, Kenya 2015 [Third:[Available from: http://www.nascop.or.ke/wp-content/uploads/2016/08/THE-KENYA-HIV-TESTING-SERVICES-GUIDELINES.pdf.

[20] De Cock KM, Barker JL, Baggaley R, El Sadr WM (2019). Where are the positives? HIV testing in sub-Saharan Africa in the era of test and treat. AIDS.

[21] Graham SM, Agutu C, van der Elst E, Hassan AS, Gichuru E, Mugo PM (2020). A Novel HIV-1 RNA Testing Intervention to Detect Acute and Prevalent HIV Infection in Young Adults and Reduce HIV Transmission in Kenya: Protocol for a Randomized Controlled Trial. JMIR Res Protoc..

[22] Ngoi CN, Price MA, Fields B, Bonventure J, Ochieng C, Mwashigadi G (2016). Dengue and Chikungunya Virus Infections among Young Febrile Adults Evaluated for Acute HIV-1 Infection in Coastal Kenya. PLoS One..

[23] Etyang AO, Munge K, Bunyasi EW, Matata L, Ndila C, Kapesa S (2014). Burden of disease in adults admitted to hospital in a rural region of coastal Kenya: an analysis of data from linked clinical and demographic surveillance systems. Lancet Glob Health.

[24] Muthumbi E, Morpeth SC, Ooko M, Mwanzu A, Mwarumba S, Mturi N (2015). Invasive Salmonellosis in Kilifi, Kenya. Clinical Infectious Diseases..

[25] Henson SP, Boinett CJ, Ellington MJ, Kagia N, Mwarumba S, Nyongesa S (2017). Molecular epidemiology of Klebsiella pneumoniae invasive infections over a decade at Kilifi County Hospital in Kenya. Int J Med Microbiol.

[26] Scott JAG, Hall AJ, Muyodi C, Lowe B, Ross M, Chohan B (2000). Aetiology, outcome, and risk factors for mortality among adults with acute pneumonia in Kenya. The Lancet.

[27] Sanders EJ, Wahome E, Powers KA, Werner L, Fegan G, Lavreys L, et al. Targeted screening of at-risk adults for acute HIV-1 infection in sub-Saharan Africa. AIDS. 2015;29(0 3):S221–30. 10.1097/QAD.0000000000000924 PMC4714928 26562811

[28] Wahome E, Fegan G, Okuku HS, Mugo P, Price MA, Mwashigadi G (2013). Evaluation of an empiric risk screening score to identify acute and early HIV-1 infection among MSM in Coastal Kenya. AIDS.

[29] Agutu CA, Oduor TH, Kombo BK, Mugo PM, Chira SM, Ogada FW (2021). High patient acceptability but low coverage of provider-initiated HIV testing among adult outpatients with symptoms of acute infectious illness in coastal Kenya. PLoS One..

[30] Sharma M, Ying R, Tarr G, Barnabas R (2015). Systematic review and meta-analysis of community and facility-based HIV testing to address linkage to care gaps in sub-Saharan Africa. Nature.

[31] Dovel K, Shaba F, Offorjebe OA, Balakasi K, Nyirenda M, Phiri K (2020). Effect of facility-based HIV self-testing on uptake of testing among outpatients in Malawi: a cluster-randomised trial. Lancet Glob Health.

[32] Sambah F, Baatiema L, Appiah F, Ameyaw EK, Budu E, Ahinkorah BO (2020). Educational attainment and HIV testing and counselling service utilisation during antenatal care in Ghana: Analysis of Demographic and Health Surveys. PLoS One..

[33] Gebregziabher M, Dai L, Vrana-Diaz C, Teklehaimanot A, Sweat M (2018). Gender disparities in receipt of HIV testing results in six sub-saharan African countries. Health equity.

[34] NACC. Kenya HIV Estimates Report 2018 [updated Oct 2018. Available from: https://nacc.or.ke/wp-content/uploads/2018/12/HIV-estimates-report-Kenya-20182.pdf.

[35] Inwani I, Chhun N, Agot K, Cleland CM, Rao SO, Nduati R, et al. Preferred HIV Testing Modalities Among Adolescent Girls and Young Women in Kenya. Journal of Adolescent Health. 2021;68(3):497-507. 10.1016/j.jadohealth.2020.07.007 32792256

[36] Singh K, Luseno W, Haney E (2013). Gender equality and education: Increasing the uptake of HIV testing among married women in Kenya. Zambia and Zimbabwe AIDS care.

[37] Leddy AM, Weiss E, Yam E, Pulerwitz J (2019). Gender-based violence and engagement in biomedical HIV prevention, care and treatment: a scoping review. BMC Public Health.

[38] Settergren SK, Mujaya S, Rida W, Kajula LJ, Kamugisha H, KilonzoMbwambo J (2018). Cluster randomized trial of comprehensive gender-based violence programming delivered through the HIV/AIDS program platform in Mbeya Region, Tanzania: Tathmini GBV study. PLoS One..

